# DNA-Membrane Anchor Facilitates Efficient Chromosome Translocation at a Distance in Bacillus subtilis

**DOI:** 10.1128/mBio.01117-19

**Published:** 2019-06-25

**Authors:** Nikolai P. Radzinski, Marina Besprozvannaya, Eric L. McLean, Anusha Talwalkar, Briana M. Burton

**Affiliations:** a Department of Bacteriology, The University of Wisconsin—Madison, Madison, Wisconsin, USA; b Department of Molecular and Cellular Biology, Harvard University, Cambridge, Massachusetts, USA; Massachusetts Institute of Technology

**Keywords:** *Bacillus*, chromosome organization, chromosome segregation, sporulation

## Abstract

Chromosome segregation in sporulating Bacillus subtilis involves the tethering of sister chromosomes at opposite cell poles. RacA is known to mediate chromosome tethering by interacting with both centromere-like elements in the DNA and with DivIVA, a membrane protein which localizes to the cell poles. RacA has a secondary function in which it assists in nucleoid condensation. Here we demonstrate that, in addition to positioning and condensing the chromosome, RacA contributes to efficient transport of DNA by the chromosome segregation motor SpoIIIE. When RacA is deleted, one-quarter of cells fail to capture DNA in the nascent spore, yet 70% of cells fail to form viable spores without RacA. This discrepancy indicates that RacA possesses a role in sporulation beyond DNA capture and condensation. We observed that the mutant cells had reduced chromosome translocation into the forespore across the entire length of the chromosome, requiring nearly twice as much time to move a given DNA locus. Additionally, functional abolition of the RacA-DivIVA interaction reduced translocation to a similar degree as in a *racA* deletion strain, demonstrating the importance of the RacA-mediated tether in translocation and chromosome packaging during sporulation. We propose that the DNA-membrane anchor facilitates efficient translocation by SpoIIIE, not through direct protein-protein contacts but by virtue of physical effects on the chromosome that arise from anchoring DNA at a distance.

## Abstract (precis)

To properly segregate their chromosomes, organisms tightly regulate the organization and dynamics of their DNA. Aspects of the process by which DNA is translocated during sporulation are not yet fully understood, such as what factors indirectly influence the activity of the motor protein SpoIIIE. In this work, we have shown that a DNA-membrane tether mediated by RacA contributes to the activity of SpoIIIE. Loss of RacA nearly doubles the time of translocation, despite the physically distinct locations these proteins and their activities occupy within the cell. This is a rare example of an explicit effect that DNA-membrane connections can have on cell physiology and demonstrates that distant changes to the state of the chromosome can influence motor proteins which act upon it.

## INTRODUCTION

Faithful chromosome segregation is vital for the propagation of all organisms. During mitosis, eukaryotic sister chromosomes are anchored to the spindle by kinetochores, which are multiprotein complexes and assemble specialized regions of chromosomes known as the centromere (1). The spindle apparatus uses the kinetochores to pull sister chromosomes apart toward opposite cell poles (2). While still poorly understood in general, in recent years, our understanding of similar processes occurring in bacterial cells has broadened (3). In rod-shaped bacteria, chromosome segregation is organized by the movement of the region of the chromosome containing the origin of replication to opposite poles (4). Several recent studies have demonstrated that chromosome segregation is at least partially entropy driven and can occur spontaneously (5, 6). Yet, certain elements of chromosome segregation require highly tuned regulation. For example, regions of chromosomes that are trapped on the wrong side of the division septum undergo further segregation by RecA-like SpoIIIE/FtsK translocases (7, 8).

SpoIIIE/FtsK translocases are recruited to the division plane and transport DNA into the correct cellular compartment (7, 9). To ensure DNA is transported to the correct location, SpoIIIE/FtsK translocases recognize short noncoding sequences distributed throughout the chromosome (10–12). Most of these sequences are oriented codirectionally (∼85%) and so guide the motor’s activity in the correct direction along the chromosome (9). This process of ensuring proper compartmentalization of DNA is known as “directional transport” (13).

During asymmetric division, such as occurs during sporulation in Bacillus subtilis, chromosome segregation assisted by SpoIIIE is vital for the production of a functional spore (14). FtsK/SpoIIIE is the only protein family identified as essential for chromosome segregation in all known bacteria (3–5). Upon capturing the origin-proximal 30% of the chromosome in the forespore, the SpoIIIE translocase moves 70% (3 Mb) of the forespore chromosome across the asymmetric septum during sporulation (15). During the initial stages of sporulation, both daughter chromosomes are anchored to the cell poles to ensure that DNA will be successfully captured within the forespore (16, 17). Early microscopic studies revealed that DNA capture is preceded by a change in morphology of the nucleoid from its normal diffuse shape (as observed during vegetative growth) to a compacted and extended form called the axial filament (16, 17).

RacA is a protein that binds centromere-like elements, known as ram (RacA binding motif) sites with its N-terminal helix-turn-helix motif (18, 19). These ram sites are found at high density near the origin of replication but are distributed throughout the rest of the chromosome (18). RacA contributes to chromosome condensation by binding specifically to the ram sites and nonspecifically elsewhere on the chromosome and oligomerizing, resulting in the axial filament structure and allowing for chromosome tethering at the cell poles (16–19). The oligomerization of RacA on the DNA into the axial filament is the result of both its C-terminal coiled coil and its N-terminal helix-turn-helix domains (19). The anchoring of sister chromosomes to the cell poles by RacA is the result of the interaction between the coiled coil domain of the curvature-localizing membrane-binding protein DivIVA and the RacA C-terminal coiled coil domain (16, 17, 19–22).

The primary roles established for RacA to date are to ensure a chromosome is localized for capture within a forespore and to contribute to the initiation of chromosome packaging for spore development (16, 17, 23, 24). Without RacA, ∼50% of cells fail to capture a chromosome with their asymmetric septa, forming anucleate forespores (16, 17). When this occurs, a backup mechanism allows cells to put down a second asymmetric septum, thereby giving cells another chance to capture DNA (16, 17). By the end of this stage of early spore development, approximately 25% of mutant cells have failed to capture a chromosome at either cell pole (17). Additionally, capture of DNA inside the forespore is necessary for proper assembly of the SpoIIIE motor at the septum (25). Here, we asked if RacA contributes to chromosome segregation beyond its role in packaging.

In this work, we demonstrate that a protein involved in chromosome positioning can impact the dynamics of a spatially distant DNA motor. We found that deleting RacA greatly impairs DNA translocation and that this effect is more apparent in SpoIIIE-deficient mutants. The translocation defect of Δ*racA* cells becomes progressively worse along the length of the chromosome, suggesting that RacA contributes to the efficient directional movement of DNA throughout chromosome segregation and not just during the initial stages. To separate the two known functions of RacA (chromosome condensation and cell pole tethering), the tethers were abolished by disrupting the RacA-DivIVA interaction. The chromosome translocation efficiency of these cells was impaired to a similar degree as in Δ*racA* cells. Together, our findings indicate that anchoring of DNA contributes to sporulation not only by localizing DNA for capture in the forespore but also via an indirect contribution to SpoIIIE translocation activity. Since the impact is not the result of direct protein-protein contacts between RacA and SpoIIIE, the effect of RacA on translocation may be due to physical changes in the chromosome that result from the polar anchor.

## RESULTS

### A discrepancy in sporulation values indicates an additional role of RacA.

Previous studies of RacA explored its impact on chromosome positioning. These studies followed sporulating cells with 1 or 2 septa and evaluated whether DNA had been misplaced and trapped outside the forespores (17). For clarity, we will henceforth refer to DNA that is successfully localized within the forespore by a septum as being “captured.” Approximately 50% of Δ*racA* cells failed to capture DNA with the first septum, and this success rate continued with the placement of a second, backup septum in the cases where the first failed (17). Those data together suggest roughly one-quarter of Δ*racA* cells failed both attempts to capture DNA in a forespore (17). The authors of that study further reported that 50% of Δ*racA* cells failed to sporulate. We sought to address the 2-fold discrepancy between the fraction of cells that failed to sporulate and the smaller fraction that had failed to capture DNA, and so began by repeating the sporulation efficiency assay.

**FIG 1:**
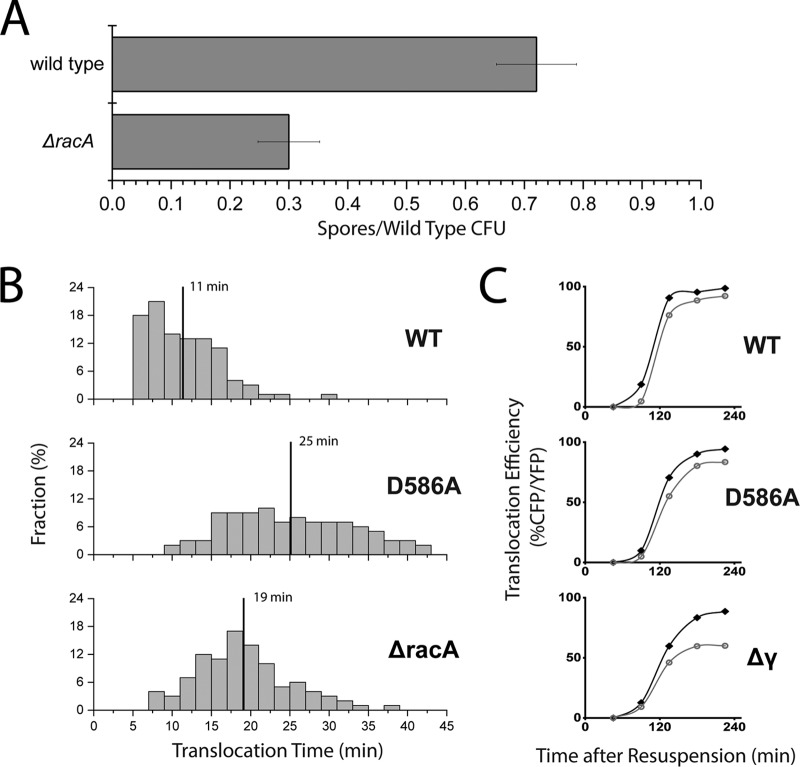
RacA contributes to sporulation by assisting SpoIIIE activity. (A) Sporulation efficiency of wild-type and Δ*racA* cells. Sporulation was induced by resuspension in minimal medium for 24 h. The 30% ± 5% efficiency for Δ*racA* cells was calculated as the number of spores as a fraction of wild-type CFU, where normalization of cell density was conducted at resuspension. Error bars are the standard deviations between dilutions; 100 to 300 colonies were counted for each condition. An efficiency of 31% ± 4% was observed by a different technique, sporulation by exhaustion. (B) Distribution of translocation times measured by live-cell time-lapse imaging of YFP and CFP signals from the forespores of sporulating cells. One hundred cells were acquired for wild type (WT), SpoIIIE^D586A^ (D586A), and Δ*racA* cells. The fraction is the proportion of the cells which saw a complete translocation event (YFP signal followed by CFP) with a given duration between the appearances of each signal. The vertical lines denote the mean translocation time in a given background, where for wild-type cells it was 11 min, SpoIIIE^D586A^ cells it was 25 min, and Δ*racA* cells it was 19 min. The *cfp* gene was at the 90° locus in each strain. (C) Translocation efficiency of wild-type (black) and Δ*racA* (gray) cells in three SpoIIIE backgrounds. DNA translocation by wild type SpoIIIE (WT), SpoIIIE^D586A^ (D586A), and SpoIIIE^Δγ^ (Δγ) of the 90° locus is shown after sporulation by resuspension; 500 to 1,000 forespores were included across 3 to 10 fields of view in technical replicates. Time points early in sporulation or with slowly sporulating strains may include 100 to 200 forespores.

We resuspended cells in minimal medium for 24 h, and colonies were plated before and after a heat kill. Colonies were also plated at the time of resuspension in order to normalize to the presporulation cell density and thus reflect the broader effects of RacA on the sporulating cell population (16, 17). These cultures were diluted before plating so that by counting colonies (100 to 300 colonies for each condition across multiple dilutions), the fractions of cells sporulating could be calculated. We observed that 30% ± 5% of Δ*racA* cells successfully sporulated when normalized to the wild-type CFU (Fig. 1A). An efficiency of 31% ± 4% was similarly observed when the cells were sporulated by a different technique, sporulation by exhaustion. In this case, approximately 500 colonies were counted for each condition across multiple dilutions. Since only 25% of Δ*racA* cells failed to capture DNA in a forespore but 70% ± 5% failed to sporulate at all, this reexamination of DNA capture versus sporulation efficiency confirmed that RacA might have some additional function contributing to sporulation. Because RacA is only present transiently early in sporulation, any impact on sporulation efficiency is likely to occur in this early window of time when DNA translocation is occurring (16).

### RacA contributes to efficient translocation of 90° locus by SpoIIIE.

To determine what other role RacA may have in sporulation, we asked if a deletion of *racA* affects DNA transport *in vivo*. To test this, we employed a previously described fluorescence assay (26). In short, a *yfp* reporter gene was integrated near the origin, which is always positioned in the forespore in sporulating wild-type cells. A *cfp* reporter gene was integrated at a region of the chromosome that is always captured in the mother cell in wild-type cells. Both reporters were placed under a promoter dependent on the forespore-specific transcription factor σ^F^ so that *yfp* and *cfp* were only expressed in the forespore. Thus, a yellow fluorescent protein (YFP) signal demonstrates that a septum has separated the mother cell from the forespore, within which the *ori-*proximal chromosome region containing *yfp* has been captured, while a cyan fluorescent protein (CFP) signal indicates that the region of the chromosome containing the *cfp* gene has been translocated from the mother cell into the forespore. This system can be used to estimate translocation rates with time-lapse microscopy by measuring the time between YFP and CFP expression in single cells or it can be used to indirectly identify changes by examining the fraction of CFP^+^/YFP^+^ cells in the population over time.

In wild-type cells, the asymmetric septum initially captures 30% of the chromosome, which spans from approximately −60° (or 300°) to 40° (on a 360° circular chromosome) (27, 28). In the absence of RacA, 50% of cells do not capture DNA within the first forespore, which causes them to try again and form a second asymmetric septum (16, 17). Those Δ*racA* cells which do successfully capture DNA in a forespore capture the *ori* region and so are properly oriented for translocation of the remaining chromosomal DNA into the forespore (9, 24, 29). Additionally, in contrast to mutant strains that lack the chromosome partitioning proteins, Soj and Spo0J, the absence of RacA does not significantly affect chromosome architecture (17, 30). To ensure we examined those cells that capture the origin in the forespore, we assayed only the cells that had YFP fluorescence in either forespore.

First, we observed the impact that a *racA* deletion has on translocation by following translocation of the *cfp* gene at the 90° locus, which is one of the first positions on the chromosome to be transported into the forespore after the formation of a division septum (27). One hundred live cells were tracked over 2 to 4 h across multiple fields of view in microfluidic chambers in order to identify the times YFP and CFP were each first detected for each individual cell (Fig. 1B). The difference between these events reflects the time of translocation from initiation of translocation until the point where the 90° locus was transported across the septum. While cells with wild-type SpoIIIE transported the 90° locus in an average of 11 min, Δ*racA* cells required an average of 19 min, nearly twice as much time. In comparison, the slowly translocating SpoIIIE variant SpoIIIE^D586A^ took 25 min, 2.5× longer than the wild type, recapitulating previous estimates derived from a population assay (26). It should be noted that previously, SpoIIIE^D586A^ was identified as SpoIIIE^D584A^, with position designations based on an early genome sequence of Bacillus subtilis (26, 32, 52, 53). Updated genome sequencing has shown that the correct position for the mutant should be SpoIIIE^D586A^ and so henceforth, we will refer to it as such (31).

To screen a wider variety of conditions, the previously described assay examining the CFP/YFP ratio over time using static time points was performed. Five hundred to 1,000 forespores were counted across 3 to 10 fields of view in technical replicates. Tracking the transport of the 90° locus by wild-type SpoIIIE cells, there was still a notable difference between cells with and without RacA (Fig. 1C). As seen both in earlier work and in the doubling of translocation time in Δ*racA* cells with the more direct single cell assay here, even small changes detected by this assay reflect significant changes to translocation timing (26, 32). The portion of Δ*racA* cells that transported the CFP reporter into the forespore was consistently below that of the wild type throughout sporulation.

To better resolve differences in DNA translocation, we used two previously described variants of SpoIIIE that exhibit slower DNA translocation rates: SpoIIIE^Δγ^ and SpoIIIE^D586A^ (Fig. 1C) (26, 32). SpoIIIE^Δγ^ is missing the DNA interacting γ domain, which is responsible for dictating the direction of DNA transport through sensing SpoIIIE recognition sequences (SRS) within the chromosome (9, 32). The γ domain couples the recognition of SRS to the regulation of ATPase activity of the motor domain, and so deleting the γ domain results in sequence insensitivity and severely impaired ATPase activity (32, 33). Thus, SpoIIIE^Δγ^ translocates DNA *in vivo* nearly 10-fold more slowly than wild-type SpoIIIE and exhibits a dramatic sporulation defect of over 4 orders of magnitude (9, 32). SpoIIIE^D586A^ is a variant that displays a 2.5-fold defect in the rate of DNA translocation compared to that of the wild type and exhibits a mild sporulation phenotype, producing ∼80% the number of spores as the wild type (26, 34). Modeling based on the structure of a homologous protein indicates that the D586A mutation is in the subunit interface between adjacent motor domains, which suggests that SpoIIIE^D586A^ may be impaired in the assembly of the functional hexamer (35). Interestingly, our single-cell translocation time data supports this further due to the broadening of the distribution of translocation times compared to that of the wild type, a likely outcome of stochastic disassembly of a functional complex (Fig. 1B). Thus, the mechanistic defects of SpoIIIE^Δγ^ and SpoIIIE^D586A^ are likely different, and so these SpoIIIE variants were attractive candidates for studying the potential effect on translocation caused by deletion of *racA*.

**FIG 2:**
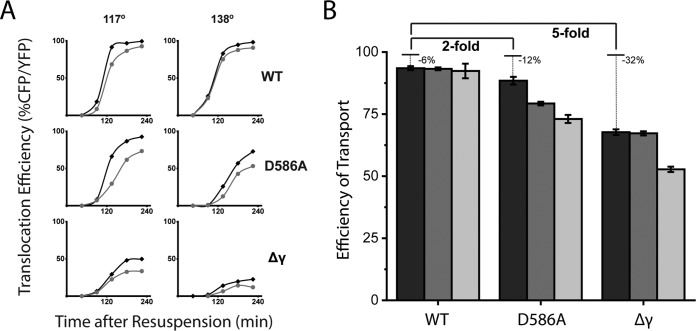
RacA contributes to DNA transport throughout the process of translocation. (A) DNA translocation of wild-type (black) and Δ*racA* (gray) cells, transporting either the 117° (left) or 138° (right) locus and either wild-type SpoIIIE (WT), SpoIIIE^D586A^ (D586A), or SpoIIIE^Δγ^ (Δγ); 500 to 1,000 forespores were included across 3 to 10 fields of view in technical replicates at each time point. Time points early in sporulation or with slowly sporulating strains may include 100 to 200 forespores. (B) Efficiency of transport for Δ*racA* cells with either wild-type SpoIIIE (WT), SpoIIIE^D586A^ (D586A), or SpoIIIE^Δγ^ (Δγ) and the CFP reporter at either the 90° (black), 117° (dark gray), or 138° (light gray) locus. Efficiency of transport is the CFP/YFP ratio of Δ*racA* normalized to wild type 225 min after the initiation of sporulation by resuspension. Data are from Fig. 2A and error bars are the standard deviations between fields of view. Fold change is relative to wild-type efficiency of transport.

Loss of RacA resulted in even greater impairment of translocation of the 90° locus in cells with both SpoIIIE^Δγ^ and SpoIIIE^D586A^ (Fig. 1C, middle and bottom). While we observed only a 5% ± 1% reduction in cells 225 min into sporulation that successfully transported the 90° locus into the forespore with wild-type SpoIIIE when *racA* was deleted, Δ*racA* cells with SpoIIIE^Δγ^ had 32% ± 1% fewer successful translocations at the same time point, a 6-fold change (Fig. 2B). Cells with SpoIIIE^D586A^ exhibited a milder defect, with 12% ± 2% fewer cells without *racA* transporting the 90° locus into the forespore. This was a 2-fold increase in the severity of the Δ*racA* defect compared to that of the wild-type SpoIIIE (Fig. 2B). As before, 500 to 1,000 forespores were counted across 3 to 10 fields of view in technical replicates. Because RacA contributes to translocation by wild-type and variant motors, the effect on transport is likely to be important for the general process of DNA movement and organization rather than one particular aspect of SpoIIIE function.

### RacA contributes to efficient DNA movement of the entire chromosome.

Since RacA contributes to the transport of the 90° locus, we asked whether the Δ*racA* DNA translocation defect extends along the length of the chromosome. To answer this question, we tracked the translocation of 117° and 138° loci, which are transported into the chromosome after the 90° locus (Fig. 2A). Again, 500 to 1,000 forespores were counted across 3 to 10 fields of view in technical replicates. The 117° and the 138° loci are ∼330 kbp and ∼590 kbp away from the 90° locus, respectively. We observed that the Δ*racA* defect is propagated along the length of the chromosome in SpoIIIE mutants (Fig. 2A). As with the 90° locus, wild-type SpoIIIE seems to translocate both loci with only a moderate defect with or without RacA (Fig. 2A, top). On the other hand, both SpoIIIE^Δγ^ and SpoIIIE^D586A^ translocate both the 117° and the 138° loci more rapidly in the presence of RacA than without it (Fig. 2A, middle and bottom). The impact of the Δ*racA* defect in transporting the 117° locus is exaggerated in comparison to the transport of the 90° locus, with 33% ± 1% and 21% ± 1% of SpoIIIE^Δγ^ and SpoIIIE^D586A^ cells, respectively, translocating this locus (Fig. 2A and B). The defect is further exaggerated for the 138° locus, with 47% ± 1% and 27% ± 2% of SpoIIIE^Δγ^ and SpoIIIE^D586A^ cells, respectively, translocating this locus (Fig. 2A and B). This progressively increasing effect demonstrates that RacA impacts DNA translocation throughout the process of chromosome segregation and not just at the chromosome capture stage (Fig. 2B).

### Abolition of the chromosomal anchoring function of RacA.

RacA has two previously described functions: chromosome anchoring and DNA condensation (16, 17). Since Δ*racA* cells exhibited a DNA translocation defect along the entire length of the chromosome, we wondered whether one or both functions of RacA contribute to efficient DNA translocation throughout the process of DNA segregation. To study this, we separated the ability of RacA to condense DNA from its ability to anchor the chromosome by deleting carboxy-terminal amino acid residues of DivIVA. Two-hybrid interaction data suggested these residues are solely responsible for interaction with RacA (16, 17, 36). DivIVA has a role in myriad cellular processes, and significant changes to its sequence result in a growth defect, a propensity to form minicells, and extensive chaining (36). The two-hybrid data suggested deleting at least 11 amino acids from the C terminus would be sufficient to ensure abolition of the RacA-DivIVA interaction while minimizing the impact on the rest of the cell (36). In case removing 11 amino acids was not sufficient to abolish the functional intermolecular interaction, we further examined the interaction in DivIVAΔ21.

**FIG 3:**
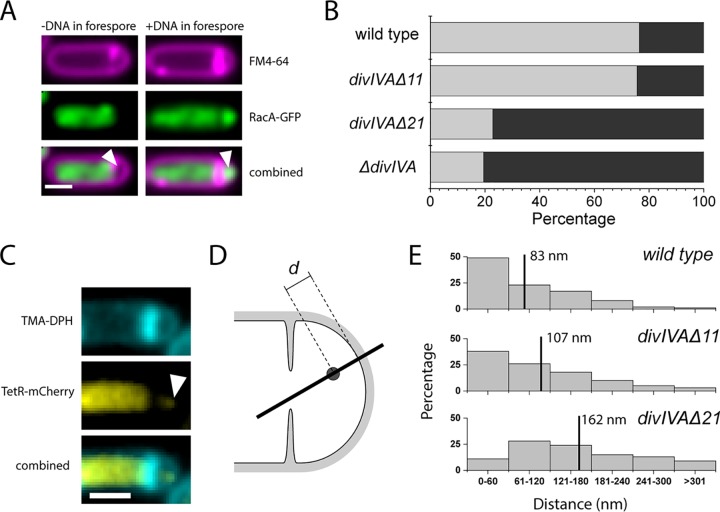
Deletion of 21 residues from the C terminus of DivIVA abolishes its interaction with RacA. (A) Sample images of RacA-GFP in wild-type cells stained with FM4-64. Bar, 1 μm. White arrowheads point to forespores empty of DNA (left) and containing DNA (right). (B) Fractions of forespores with (gray) and without (black) detectable RacA-GFP fluorescence 180 min after sporulation by resuspension. At least 500 forespores were counted for each strain. (C) Sample images of TetR-mCherry in wild-type cells stained with TMA-DPH and used for line scans. Bar, 1 μm. The white arrowhead denotes a TetR-mCherry focus. (D) Diagram of line scan (black line) through a forespore, intersecting a TetR-mCherry focus (black circle) and the closest region of TMA-DPH-stained membrane. These line scans were used for calculating the distance *d* between the locus and the membrane. (E) Distributions of distances calculated between each focus and the nearest portion of the membrane. For wild-type cells, the mean distance was 83 nm (upper black vertical line) and for DivIVAΔ11 cells, the mean was 107 nm, while for DivIVAΔ21 cells, the mean was 162 nm (lower black vertical line). One hundred line scans were performed for each strain.

To evaluate whether the RacA-DivIVA interaction was abolished in DivIVAΔ11 and DivIVAΔ21, we quantified DNA capture in the forespore of DivIVAΔ11 and DivIVAΔ21 cells by imaging fluorescently tagged RacA-green fluorescent protein (GFP) (Fig. 3A). All data were collected 3 h after induction of sporulation by resuspension, and at least 500 forespores were counted for each strain. RacA-GFP fluorescence was not detected in 24% of wild-type or DivIVAΔ11 forespores (Fig. 3A). In contrast, 77% of *divIVA*Δ21 forespores failed to capture chromosomes by this assay. As expected, we saw the same defect in Δ*divIVA* cells, where there should be no interaction. The discrepancy between these data and previous work indicating all wild-type cells and half of Δ*racA* cells fail to capture DNA is likely due to our use of a particularly stringent threshold of fluorescence detection (16). To get a more precise and independent measure of the RacA-DivIVA interaction, we took an approach to more directly visualize the tether in a system with wild-type RacA.

To further verify the RacA-DivIVA interactions were abolished in DivIVAΔ21 cells, we directly visualized the intracellular position of a specific *ori*-proximal locus with respect to the cell membrane (Fig. 3B and C). We inserted an array of Tet repressor binding sites (*tetO*) into the chromosome at the *yycR* locus where the ram sites targeted by RacA are at a high density. TetR-mCherry was expressed under a xylose-inducible promoter, and distinct foci could be visualized. One hundred foci were evaluated for each strain. The position of a given TetR-mCherry focus was determined by aligning a line scan with the closest portion of TMA-DPH [1-(4-trimethylammoniumphenyl)-6-phenyl-1,3,5-hexatriene *p*-toluene sulfonate]-stained membrane and fitting a window of pixels around the peak intensity to a Gaussian distribution (Fig. 3B). A Gaussian fit of the pixels around the peak TMA-DPH intensity on the line scan provided the membrane position so that the distance between the focus and the cell membrane could be measured.

We observed that wild-type cells had a mean distance of 83 nm between foci and the membrane (Fig. 3C, top). The distribution of distances was similar in DivIVAΔ11 cells, with a mean distance of 107 nm (middle). Cells containing *divIVA*Δ21, however, had a mean distance of 162 nm between foci and the membrane (bottom). The near doubling in mean distance of the focus from the membrane between wild-type and *divIVA*Δ21 cells suggests that the RacA-DivIVA interaction is functionally abolished in DivIVAΔ21 cells. While RacA-GFP localization and TetR-mCherry localization are indirect measurements of the DivIVA-RacA interaction, taken together with the earlier two-hybrid data, they strongly indicate that the functional interaction has been effectively abolished in DivIVAΔ21 to the point where the tether itself can be satisfactorily described as no longer present.

### Chromosome anchoring versus condensation.

**FIG 4:**
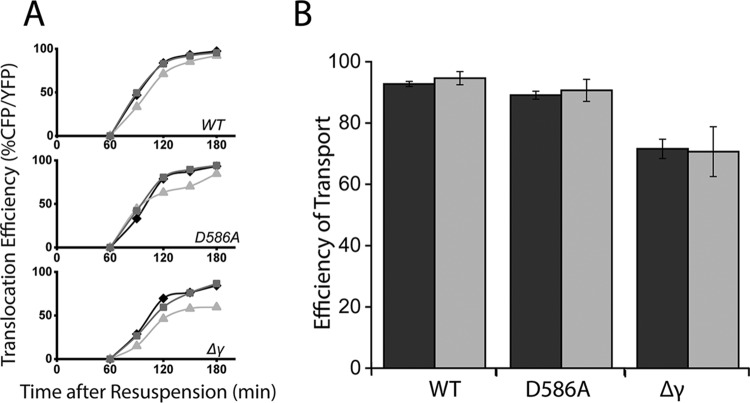
Abolition of anchoring by RacA nullifies its contribution to DNA translocation activity by SpoIIIE. (A) DNA translocation efficiency in wild-type and DivIVAΔ21 cells after sporulation by resuspension. (Top) Wild-type SpoIIIE cells (WT); (middle) SpoIIIE^D586A^ cells (D586A); (bottom) SpoIIIE^Δγ^ cells (Δγ). Wild type (black), *divIVA*Δ11 (dark gray), and *divIVA*Δ21 (light gray) are each depicted. The *cfp* reporter gene is at the 90° locus; 500 to 1,000 forespores were included across 3 to 10 fields of view in technical replicates at each time point. Time points early in sporulation or with slowly sporulating strains may include 100 to 200 forespores. (B) The efficiency of transport of Δ*racA* cells (black) compared to that of *divIVA*Δ21 cells (gray) with wild-type SpoIIIE, SpoIIIE^D586A^, and SpoIIIE^Δγ^. The efficiencies for Δ*racA* and *divIVA*Δ21 were standardized to wild-type RacA and wild-type DivIVA, respectively, 180 min after sporulation by resuspension. The data depicted are the same as in the translocation efficiency plots in Fig. 1B and 4A, and error bars are the standard deviations between fields of view. The *cfp* gene was at the 90° locus in each strain.

Having established that anchoring is functionally abolished in DivIVAΔ21 cells where RacA remains intact, we could now specifically explore the effect of chromosome anchoring on DNA translocation. We examined DivIVAΔ21 cells with the YFP-CFP assay described previously, again evaluating 500 to 1,000 forespores at each time point (Fig. 4A and B). As described above, cells contained either wild-type SpoIIIE (top), SpoIIIE^D586A^ (middle), or SpoIIIE^Δγ^ (bottom). When *cfp* was located at the 90° locus, translocation efficiency reduced by 5% ± 2% in wild-type SpoIIIE cells with DivIVAΔ21 180 min into sporulation. This is close to the 7% ± 1% reduction observed in Δ*racA* cells. Translocation efficiency was indistinguishable between wild-type and DivIVAΔ11 cells. The impact of the defect in DivIVAΔ21 cells and Δ*racA* cells was likewise similar in SpoIIIE^Δγ^ and SpoIIIE^D586A^ backgrounds. Taken together, these data indicate anchoring of DNA to the membrane by RacA contributes to SpoIIIE activity and that any contribution of DNA compaction is negligible in comparison.

## DISCUSSION

The data presented here indicate that the chromosome tethering activity of RacA contributes to DNA translocation by SpoIIIE. We show that RacA has an additional role beyond capturing DNA in the nascent forespore and condensing the chromosome. The tether anchors DNA to the membrane and provides a long-range contribution to efficient chromosome segregation.

The translocation defect for wild-type SpoIIIE in a Δ*racA* background became more apparent in the context of variants, SpoIIIE^Δγ^ and SpoIIIE^D586A^. This apparently milder impact of Δ*racA* on wild-type SpoIIIE activity is likely because the wild-type motor translocates DNA with a velocity near the limit of time resolution of the *in vivo* fluorescence DNA transport assay employed here. In fact, small deficiencies identified with this assay actually indicate significant reductions in translocation time, as seen in the live-cell quantification of translocation time, where Δ*racA* nearly doubled the time it took to transport DNA (Fig. 1B). The effects on the activity of the motor observed here in assays of translocation are significant not just by their magnitude but further by the nature of the impact. The tether is spatially separated by as much as hundreds of nanometers from the motor complex and yet assists the motor throughout chromosome translocation.

Interestingly, the impacts of SpoIIIE^D586A^ and Δ*racA* on sporulation were relatively similar. SpoIIIE^D586A^ took 2.5-fold longer than SpoIIIE to complete translocation and had a 6% reduction in CFP/YFP at the 90° locus 180 min after initiation of sporulation, while Δ*racA* cells took 2-fold longer and had a 7% reduction in CFP/YFP at the same time point (Fig. 1B and C). SpoIIIE^D586A^ cells had a sporulation efficiency defect of ∼80% that of the wild type, most likely stemming from this reduction in translocation rate (26, 34). The discrepancy between the measured sporulation efficiency and the rate of capture of DNA in Δ*racA* cells which initially inspired this work may in fact be explained by this similar reduction in translocation rate. The timing of sporulation, particularly at the time of translocation, is highly well regulated. Movement of sporulation regulators along the length of the chromosome can reduce sporulation efficiency by up to an order of magnitude (26, 37, 38). Yet in these cases, the change in timing for the regulator to move into the forespore increases by, at most, one-third. In contrast, the *racA* deletion doubles translocation time.

There have been numerous previous cases where even relatively small effects on sporulation efficiency can actually reflect significant roles in sporulation. For example, a recent transposon screen for Bacillus subtilis sporulation factors still missed 15 of 148 known genes with roles in sporulation, despite identifying several new factors (39). Several early screens even missed RacA entirely, despite its roles in chromosome capture, SpoIIIE complex nucleation, and efficient chromosome transport (16, 25, 40–42).

A number of forespores in Δ*racA* cells expressed CFP, but not YFP, and these were excluded from the YFP/CFP fraction calculations, as the lack of YFP suggested that the chromosomes had been improperly captured in the forespore. We found that ∼10% of forespores expressing CFP did not also have a detectable YFP signal in wild-type SpoIIIE cells with the *cfp* gene located at the 90° locus (see Table S1 in the supplemental material). This number aligns with results from a previous study in which fluorescently labeled chromosomal loci were localized in order to identify which regions of the chromosome had been captured in the forespore in a nontranslocating SpoIIIE background (27, 29). Those results found that ∼10% of Δ*racA* cells which successfully captured DNA in the forespore had failed to capture *ori* (29). We saw that the further from *ori* the *cfp* gene was located, the less often it was expressed without an accompanying YFP signal. This is likely because the greater distance makes it more improbable for the region to be mistakenly captured in the forespore (Table S1). Interestingly, the CFP-only fraction also decreased as the severity of the SpoIIIE defect increased, such that cells with wild-type SpoIIIE were the most likely to mistakenly express CFP alone. It is unclear why this population decreases, but it could be related to the specific mechanistic defects of SpoIIIE^D586A^ and SpoIIIE^Δγ^ as well as the organization of the chromosome at the septum.

10.1128/mBio.01117-19.1 TABLE S1 Fraction of forespores with CFP signal detected without accompanying YFP signal. Download Table S1, DOCX file, 0.1 MB. Copyright © 2019 Radzinski et al. 2019 Radzinski et al. This content is distributed under the terms of the Creative Commons Attribution 4.0 International license.

After the RacA-DivIVA interaction was abolished by deleting 21 residues from the C-terminal end of DivIVA, we observed a significant reduction in the number of forespores successfully capturing chromosomes compared to that for cells with wild-type DivIVA (Fig. 3A). We provided more direct evidence for the abolition of tethering in mutant *divIVA* cells by fluorescently labeling an origin-proximal locus. The distance between the fluorescent focus and the membrane in forespores increased from 83 nm in the wild type to 162 nm in *divIVA*Δ21 cells (Fig. 3C). The greater distance between DNA and membrane indicates it is more likely the DNA is not directly attached to the cell poles and is instead free to diffuse elsewhere in the forespore. Because the microscope images are merely two-dimensional (2D) projections of a three-dimensional (3D) structure, any information regarding distances along the *z* axis is lost, and the values measured are not absolute but relative distances. Additionally, assuming a 600-nm-diameter forespore, a freely diffusing particle will appear an average of 174 nm away from the membrane in a one-dimensional (1D) line scan. Although previous studies proposed that a Soj-mediated DNA-membrane interaction could exist in sporulating cells, the small difference between the measured average distance of 162 nm and the predicted 174 nm could indicate that, even in the absence of RacA, these Soj-mediated interactions do not play a significant role (29).

DivIVAΔ11 cells surprisingly did not show mislocalization of RacA-GFP despite the indication from the earlier two-hybrid experiments that this may be sufficient to abolish the interaction with RacA (Fig. 3B) (36). However, the localization of the TetR-mCherry focus in DivIVAΔ11 cells to an average of 107 nm from the membrane is slightly greater than the 83 nm measured in wild-type cells, indicating that, while the tether may be somewhat impaired, it is still at least transiently present (Fig. 3C). Because the RacA-GFP localization indicates DNA is still captured as successfully as in wild-type cells and the translocation efficiency of DivIVAΔ11 cells is indistinguishable from that of wild-type cells, the tethers have not been actually functionally abolished (Fig. 4A). The discrepancy between the data indicating there is some abolition of activity (the two-hybrid assay and the distance between a DNA locus and the membrane) and the data indicating there is no change (the RacA-GFP localization and YFP/CFP assays) suggests that even a reduced interaction is sufficient for maintaining the tether.

While we now understand that DNA-membrane tethers indirectly contribute to SpoIIIE activity, the precise mechanism of this effect remains unclear. There are only a few other examples of DNA-membrane interactions in bacteria to compare to (43). DNA-membrane tethers have been hypothesized to change the general architecture of the chromosome by providing an expansion force to contrast the myriad compacting forces which condense the volume of the nucleoid (44). This expansion force is believed to provide macromolecules the ability to freely interact with the chromosome (43). The Δ*racA* defect we observed persisted regardless of the SpoIIIE background and throughout the process of translocation. This combined with the distance between the tether and the SpoIIIE complex and the lack of known partners that could facilitate a direct interaction between RacA and SpoIIIE suggests that the tether produces a global physical effect. One possibility that will be a subject of future studies is that the tether may reorganize the chromosome itself.

A possible explanation for how a tether could influence translocation is by providing an expansion force for the chromosome. Because of the high rate at which SpoIIIE translocates, it may be important for the DNA polymer to remain mobile in order to be drawn toward the SpoIIIE complex as quickly as it is pumped through it. By sequestering the *ori*-proximal region of the chromosome to the pole of the forespore, the chromosome should more efficiently fill the volume available, as the tether would provide a counter to the many compacting forces that reduce DNA mobility (43, 44). Proper packaging of DNA into the forespore is important, particularly as the forespore swells with DNA through sporulation (45). However, SpoIIIE has no problem stripping off large quantities of DNA-bound protein at a high rate during translocation, and so it is not yet obvious how sensitive the motor is to local or global DNA structure (34). Additionally, the other role of RacA in compacting the chromosome appears to contradict the idea that it would provide an expansion force. Regardless of the specific mechanism, the effects of DNA-membrane tethering by RacA on the chromosome are likely to be large scale and suggest that even a strong motor such as SpoIIIE may be influenced by long-range interactions and the global state of cellular DNA.

## MATERIALS AND METHODS

### Bacterial growth conditions.

All B. subtilis strains are given in Table S2 and all primers are given in Table S3 in the supplemental material. Strains were derived from laboratory prototrophic strain PY79 (46). Transformation of B. subtilis was performed with double-stranded PCR fragments, B. subtilis genomic DNA, or linearized plasmid (47). Synchronized sporulation was induced by resuspension in minimal medium at *A*_600_ of 0.6 at 37°C (48). All plasmids were propagated in Escherichia coli strain DH5α, which was grown and transformed as previously described (49). TetR-mCherry-containing strains were maintained in 40 ng/ml anhydrous tetracycline (aTc) in order to inhibit TetR-mCherry binding to *tetO* sites. Additionally, 6% (wt/vol) xylose was added to the medium 60 min before resuspension into minimal medium to induce expression of TetR-mCherry. Xylose and aTc were each not included in minimal medium after resuspension. Cell membranes were stained with either 50 μM TMA-DPH or 2.5 μM FM4-64 in 1× phosphate-buffered saline (PBS). Sporulation efficiency assays were performed as previously described (47).

10.1128/mBio.01117-19.2 TABLE S2 Strains used in this study. Download Table S2, DOCX file, 0.1 MB. Copyright © 2019 Radzinski et al. 2019 Radzinski et al. This content is distributed under the terms of the Creative Commons Attribution 4.0 International license.

10.1128/mBio.01117-19.3 TABLE S3 Primers used in this study. Download Table S3, DOCX file, 0.1 MB. Copyright © 2019 Radzinski et al. 2019 Radzinski et al. This content is distributed under the terms of the Creative Commons Attribution 4.0 International license.

### Cloning.

For two-color DNA transport assay, long-flanking PCR was performed to insert P_IIQ_-*cfp* (*tet*) into *yhdGH* (90°), *ykcC* (117°), and *ylyA* (138°). All clones were propagated in E. coli DH5α cells and verified by sequencing. Oligonucleotides and strain information are provided in the supplemental material.

### Image analysis.

Image analysis was performed with FIJI (Fiji Is Just ImageJ) (50). CFP and YFP experiment quantification was performed manually. During identification of both YFP^+^ and CFP^+^ forespores, the minimum display range was set as low as possible so as to include any detectable signal, and any forespore twice as bright as the noise above background fluorescence was counted. For live-cell time-lapse experiments, the first time point YFP or CFP was detected was noted for each cell, and the difference between the two values was calculated. For bulk experiments with static time points, forespores with YFP expression were identified and counted. Then, forespores with YFP and CFP expression were identified and counted. Five hundred to 1,000 forespores were included for most time points, with multiple fields of view included for each sample. Some early time points and strains with sporulation defects had smaller numbers (*n* = 100 to 200).

For TetR-mCherry focus colocalization with the membrane, cells which contained a forespore visible with the TMA-DPH stain were identified after a single iteration of the FIJI smoothing function was applied. If the forespore had exactly one TetR-mCherry focus, a 2D line scan was made between the focus and the closest region of the membrane. The position of the focus was identified by fitting the peak intensity pixel and a 7-pixel-wide window around it to a Gaussian distribution where $y=A\;e^{-{\left(x-a\right)}^{2}/2b^{2}}$ and *a* is the center of the peak. The closest TMA-DPH fluorescence intensity peak from here on the line scan was then identified and similarly fit to a Gaussian distribution. The distance was calculated between these two subpixel positions. One hundred line scans were performed for each experiment, collected across several individual fields of view. Images included in figures underwent a single iteration of the FIJI smoothing function.

### Fluorescence microscopy.

Fluorescence microscopy was performed on an inverted Zeiss Observer.Z1 and an upright Zeiss Imager.M1, each with a Photometrics Cool*SNAP* HQ2 camera. The data were collected on the upright microscope as previously described (26). Samples stained with TMA-DPH were imaged on the inverted microscope (this includes some images of the CFP/YFP translocation assay in containing *divIVA* variants in Fig. 4 as well as all images of the TetR-mCherry foci in Fig. 3). Fluorescence was activated with a Zeiss Colibri light-emitting diode (LED) light source. TMA-DPH was activated with a 353-nm LED for 1 s at 100% source intensity. TetR-mCherry was activated with a 589-nm LED for 2 to 10 s at 100% source intensity. Images were acquired with the Zeiss ZEN 2.3 software package. All white-light images were obtained using phase-contrast microscopy.

For time-lapse imaging, images were acquired every 2 min with tiling, definite focus, and a constant flow of medium through a fabricated polydimethylsiloxane (PDMS) microfluidic chamber. Depleted medium was prepared by sporulating wild-type PY79 cells by resuspension, spinning down the culture 2.5 h into sporulation, and filtering the supernatant in a 0.2-μm filter. Cells were adhered to the glass surface of the microfluidic device by incubating for 10 to20 min with 0.03% (wt/vol) chitosan, which had been freshly dissolved in 0.1 M acetic acid several hours before addition to the device. The chambers were then rinsed with water and depleted sporulation medium before cells were added (51). Heavy flow was applied to flatten cells onto the surface, and a constant flow of depleted medium maintained sporulation conditions. Time-lapse images were taken over 2 to 4 h, where every 2 min, phase-contrast, YFP, and CFP snapshots were taken. Phase contrast used a 20-ms exposure with a 6.4 V TL halogen lamp, YFP was activated with a 505-nm LED at 10% intensity for 1 s, and CFP was activated with a 445-nm LED at 10% intensity for 1 s.
