# Supplementary material for: DNA-Membrane Anchor Facilitates Efficient Chromosome Translocation at a Distance in Bacillus subtilis
Source: mBio. 2019 Jun 25;10(3):e01117-19. doi: 10.1128/mBio.01117-19 (PMC6593407; doi:10.1128/mBio.01117-19)
Supplement: TABLE S1 [file mBio.01117-19-st001.docx]

|  | ***cfp* at 90° locus** | | | ***cfp* at 117° locus** | | | ***cfp* at 138° locus** | | |
| --- | --- | --- | --- | --- | --- | --- | --- | --- | --- |
| **T(min)** | **SpoIIIE** | **SpoIIIE^D586A^** | **SpoIIIE^Δγ^** | **SpoIIIE** | **SpoIIIE^D586A^** | **SpoIIIE^Δγ^** | **SpoIIIE** | **SpoIIIE^D586A^** | **SpoIIIE^Δγ^** |
| **135** | 8±0.8% | 4±0.6% | 1±0.1% | 6±0.8% | 2±0.5% | 2±0.3% | 4±0.3% | 4±0.3% | 2±0.1% |
| **180** | 10±0.9% | 6±0.6% | 1±0.2% | 6±0.3% | 2±0.2% | 2±0.2% | 3±0.3% | 0±0.0% | 0±0.0% |
| **225** | 11±1.0% | 8±1.2% | 1±0.0% | 6±0.9% | 2±0.3% | 1±0.1% | 6±0.2% | 2±0.3% | 1±0.1% |

**Table S1-** Fraction of forespores with CFP signal detected without accompanying YFP signal. Time points before 135 minutes after sporulation by resuspension were not included as the number of forespores with CFP was already so low that an accurate fraction could not be calculated. Even at later times points, fewer than 100 forespores were counted. These are the same data as were included in Figures 1*B* and 2*A*.
