# Supplementary material for: DNA-Membrane Anchor Facilitates Efficient Chromosome Translocation at a Distance in Bacillus subtilis
Source: mBio. 2019 Jun 25;10(3):e01117-19. doi: 10.1128/mBio.01117-19 (PMC6593407; doi:10.1128/mBio.01117-19)
Supplement: TABLE S2 [file mBio.01117-19-st002.docx]

| **Strain** | **Genotype** | **Figure** | **Reference** |
| --- | --- | --- | --- |
| bBB906 | yycR::tetO(120) (cat) amyE::Pxyl-tetR-mCherry (spec) | 3 | this work |
| bBB907 | divIVA::divIVAΔ21aa erm, yycR::tetO(120) (cat) amyE::Pxyl-tetR-mCherry (spec) | 3 | this work |
| bBB908 | spoIIIE::neo yycR::PIIQ-yfp (phleo) ycgO::spoIIIE (cat) yhdGH::PIIQ-CFP (tet) divIVA::divIVAΔ21 (erm) | 4 | this work |
| bBB909 | spoIIIE::neo yycR::PIIQ-yfp (phleo) ycgO::spoIIIEΔγ (cat) yhdGH::PIIQ-CFP (tet) divIVA::divIVAΔ21 (erm) | 4 | this work |
| bBB910 | spoIIIE::neo yycR::PIIQ-yfp (phleo) ycgO::spoIIIED586A (cat) yhdGH::PIIQ-CFP (tet) divIVA::divIVAΔ21 (erm) | 4 | this work |
| bBB911 | racA-gfp spec | 2, S1 | Ben-Yehuda et al, 2003 |
| bBB912 | racA-gfp spec, divIVA::divIVAΔ21 (erm) | 2, S1 | this work |
| bBB913 | racA-gfp spec, divIVA::erm | 2, S1 | this work |
| bBB914 | spoIIIE::neo yycR::PIIQ-yfp (phleo) ycgO::spoIIIE (cat) ylyA::PIIQ-CFP (tet) | 2 | this work |
| bBB915 | spoIIIE::neo yycR::PIIQ-yfp (phleo) ycgO::spoIIIEΔγ (cat) ylyA::PIIQ-CFP (tet) | 2 | this work |
| bBB916 | spoIIIE::neo yycR::PIIQ-yfp (phleo) ycgO::spoIIIE D586A (cat) ylyA::PIIQ-CFP (tet) | 2 | this work |
| bBB917 | spoIIIE::neo yycR::PIIQ-yfp (phleo) ycgO::spoIIIE (cat) ykcC::PIIQ-CFP (tet) | 2 | this work |
| bBB918 | spoIIIE::neo yycR::PIIQ-yfp (phleo) ycgO::spoIIIEΔγ (cat) ykcC::PIIQ-CFP (tet) | 2 | this work |
| bBB919 | spoIIIE::neo yycR::PIIQ-yfp (phleo) ycgO::spoIIIE D586A (cat) ykcC::PIIQ-CFP (tet) | 2 | this work |
| bBB920 | racA::spec | 1 | this work |
| bBB921 | spoIIIE::neo yycR::PIIQ-yfp (phleo) ycgO::spoIIIE (cat) yhdGH::PIIQ-CFP (tet) | 1,4 | this work |
| bBB922 | spoIIIE::neo yycR::PIIQ-yfp (phleo) ycgO::spoIIIEΔγ (cat) yhdGH::PIIQ-CFP (tet) | 1,4 | this work |
| bBB923 | spoIIIE::neo yycR::PIIQ-yfp (phleo) ycgO::spoIIIE D586A (cat) yhdGH::PIIQ-CFP (tet) | 1,4 | this work |
| bBB924 | spoIIIE::neo yycR::PIIQ-yfp (phleo) ycgO::spoIIIE (cat) ylyA::PIIQ-CFP (tet) racA::spec | 2 | this work |
| bBB925 | spoIIIE::neo yycR::PIIQ-yfp (phleo) ycgO::spoIIIEΔγ (cat) ylyA::PIIQ-CFP (tet) racA::spec | 2 | this work |
| bBB926 | spoIIIE::neo yycR::PIIQ-yfp (phleo) ycgO::spoIIIE D586A (cat) ylyA::PIIQ-CFP (tet) racA::spec | 2 | this work |
| bBB927 | spoIIIE::neo yycR::PIIQ-yfp (phleo) ycgO::spoIIIE (cat) ykcC::PIIQ-CFP (tet) racA::spec | 2 | this work |
| bBB928 | spoIIIE::neo yycR::PIIQ-yfp (phleo) ycgO::spoIIIEΔγ (cat) ykcC::PIIQ-CFP (tet) racA::spec | 2 | this work |
| bBB929 | spoIIIE::neo yycR::PIIQ-yfp (phleo) ycgO::spoIIIE D586A (cat) ykcC::PIIQ-CFP (tet) racA::spec | 2 | this work |
| bBB930 | spoIIIE::neo yycR::PIIQ-yfp (phleo) ycgO::spoIIIE (cat) yhdGH::PIIQ-CFP (tet) racA::spec | 1,4 | this work |
| bBB931 | spoIIIE::neo yycR::PIIQ-yfp (phleo) ycgO::spoIIIEΔγ (cat) yhdGH::PIIQ-CFP (tet) racA::spec | 1,4 | this work |
| bBB932 | spoIIIE::neo yycR::PIIQ-yfp (phleo) ycgO::spoIIIE D586A (cat) yhdGH::PIIQ-CFP (tet) racA::spec | 1,4 | this work |
| bBB933 | racA-gfp spec, divIVA::divIVAΔ11 (erm) | 2, S1 | this work |
| bBB934 | divIVA::divIVAΔ11 (erm), yycR::tetO(120) (cat) amyE::Pxyl-tetR-mCherry (spec) | 3 | this work |
| bBB935 | spoIIIE::neo yycR::PIIQ-yfp (phleo) ycgO::spoIIIE (cat) yhdGH::PIIQ-CFP (tet) divIVA::divIVAΔ11 (erm) | 4 | this work |
| bBB936 | spoIIIE::neo yycR::PIIQ-yfp (phleo) ycgO::spoIIIEΔγ (cat) yhdGH::PIIQ-CFP (tet) divIVA::divIVAΔ11 (erm) | 4 | this work |
| bBB937 | spoIIIE::neo yycR::PIIQ-yfp (phleo) ycgO::spoIIIED586A (cat) yhdGH::PIIQ-CFP (tet) divIVA::divIVAΔ11 (erm) | 4 | this work |

**Table S2 –** The strains used in this study.
