# Supplementary material for: DNA-Membrane Anchor Facilitates Efficient Chromosome Translocation at a Distance in Bacillus subtilis
Source: mBio. 2019 Jun 25;10(3):e01117-19. doi: 10.1128/mBio.01117-19 (PMC6593407; doi:10.1128/mBio.01117-19)
Supplement: TABLE S3 [file mBio.01117-19-st003.docx]

| **Oligos** | **Sequence** | **Role** |
| --- | --- | --- |
| oMB371 | GAACTGCAGGCGTTTAACGTC | upstream DivIVA |
| oMB372 | ctacaccgccataaactatcatatcgtcttaCTCAAGGAGATGATCCCAATCG | C-terminal DivIVAΔ11aa (+erm) |
| oMB373 | ccggaggtgtagcatgtctcATTCTCTGATTATCTTGACATTTTCTTAGCTTG | downstream DivIVA |
| oMB374 | CTTTTCCGTCTTTTACACCAAAAGC | downstream DivIVA |
| oEM35 | ctacaccgccataaactatcatatcgtcttaTTTCAGAAGATCAAGCTGAGCTTCAATC | C-terminal DivIVAΔ21 (+erm) |
| oEM41 | GAGACATGCTACACCTCCGG | erm cassette |

**Table S3 –** The primers used in this study.
